# Effect of Combined Intraosseous and Intraarticular Infiltrations of Autologous Platelet-Rich Plasma on Subchondral Bone Marrow Mesenchymal Stromal Cells from Patients with Hip Osteoarthritis

**DOI:** 10.3390/jcm11133891

**Published:** 2022-07-04

**Authors:** Payal Ganguly, Nicolás Fiz, Maider Beitia, Heather E. Owston, Diego Delgado, Elena Jones, Mikel Sánchez

**Affiliations:** 1 Leeds Institute of Rheumatic and Musculoskeletal Medicine, University of Leeds, Leeds LS9 7JT, UK; p.ganguly@leeds.ac.uk (P.G.); h.e.owston@leeds.ac.uk (H.E.O.); 2 Arthroscopic Surgery Unit, Hospital Vithas Vitoria, Beato Tomás de Zumarraga 10, 01008 Vitoria-Gasteiz, Spain; nicolas.fiz@ucatrauma.com; 3 Advanced Biological Therapy Unit, Hospital Vithas Vitoria, Beato Tomás de Zumarraga 10, 01008 Vitoria-Gasteiz, Spain; maider.beitia@ucatrauma.com (M.B.); diego.delgado@ucatrauma.com (D.D.)

**Keywords:** osteoarthritis (OA), platelet rich plasma (PRP), mesenchymal stromal cells (MSCs), hip OA, senescence, ageing

## Abstract

Osteoarthritis (OA) is a debilitating condition that significantly impacts its patients and is closely associated with advancing age and senescence. Treatment with autologous platelet rich plasma (PRP) is a novel approach that is increasingly being researched for its effects. Subchondral bone mesenchymal stromal cells (MSCs) are key progenitors that form bone and cartilage lineages that are affected in OA. This study investigated the changes in subchondral bone MSCs before and after combined intraosseous (IO) and intraarticular (IA) PRP infiltration. Patient bone marrow aspirates were collected from 12 patients (four male, eight female) aged 40–86 years old (median 59.5). MSCs were expanded in standard media containing human serum to passage 1 and analysed for their colony-forming potential, senescence status, and gene expression. Hip dysfunction and Osteoarthritis Outcome Score (HOOS) at baseline and 6 months post second infiltration were used to assess the clinical outcomes; seven patients were considered responders and five non-responders. The number of colony-forming MSCs did not increase in the post treatment group, however, they demonstrated significantly higher colony areas (14.5% higher compared to Pre) indicative of enhanced proliferative capacity, especially in older donors (28.2% higher). Senescence assays also suggest that older patients and responders had a higher resistance to senescent cell accumulation. Responder and non-responder MSCs tended to differ in the expression of genes associated with bone formation and cartilage turnover including osteoblast markers, matrix metalloproteinases, and their inhibitors. Taken together, our data show that in hip OA patients, combined IO and IA PRP infiltrations enhanced subchondral MSC proliferative and stress-resistance capacities, particularly in older patients. Future investigation of the potential anti-ageing effect of PRP infiltrations and the use of next-generation sequencing would contribute towards better understanding of the molecular mechanisms associated with OA in MSCs.

## 1. Introduction

In recent years, intraarticular (IA) infiltrations of platelet-rich plasma (PRP) have emerged as an alternative to current treatments for knee osteoarthritis (OA) [1,2]. These biological and autologous therapies use the patient’s own plasma and growth factors derived from platelets, and are often combined with an autologous fibrin scaffold for regenerative purposes. These growth factors help restore joint homeostasis, have inductive and protective effects on chondrocytes, and act on the synoviocytes of the synovial membrane by stimulating the production of hyaluronic acid (HA) and other molecules. In addition, this cocktail of growth factors also has anti-inflammatory and bacteriostatic characteristics, and modulate mesenchymal stromal cells (MSCs), which are known to participate in the regeneration of cartilage. All of these properties contribute to promoting a biological environment that slows down cartilage degeneration and relieves clinical symptoms [3].

The success of this treatment does not only lie in the characteristics of the PRP [4], but also in its correct application. Although IA infiltrations reach the cartilage and the synovial membrane, this route of administration does not reach the deeper layers of the subchondral bone, thus limiting its therapeutic potential. Subchondral bone is a key element in both maintaining the homeostasis of the joint and in the pathophysiology of OA due to its direct communication with the cartilage [5,6]. The subchondral bone undergoes marked alterations during OA including an increase in the cortical plate thickness along with changes in the subchondral cortical bone mass as well as the architecture, depending upon the stage and severity of OA progression [5]. Adding intraosseous (IO) PRP injections to target subchondral bone can provide a more comprehensive treatment by stimulating biological processes that lead to an environment primed for regeneration. Restoring joint homeostasis also influences the behaviour of MSCs that coordinate bone remodelling of the subchondral bone [7].

Indeed, treating severe knee OA with a combination of IO and IA infiltrations of PRP have shown superior clinical outcomes at 6 and 12 months compared to IA injections of PRP without IO infiltration. In addition, there was a significant reduction of MSCs in the synovial fluid (SF) after treatment [8]. This decrease was not observed when patients received only IA injections, suggesting a biological effect on subchondral bone by means of IO infiltrations of PRP [9]. In another observational study, the combination of IA and IO infiltrations exerted significant pain reduction and improvement in knee joint functionality at 6 and 12 months after treatment in patients with severe knee OA [10]. Those patients treated only with IA PRP did not show this improvement [11]. In an independent study, the benefit provided by IO infiltration combined with IA injection of PRP to treat mild and moderate knee joint degeneration was compared with an IA injection of PRP and of HA, and the combination IO with IA injections of PRP resulted in superior clinical outcomes, with sustained improvement in quality of life within 18 months after the treatment [12]. This technique was also developed for applications in hip OA [11]. 

Despite the good clinical response of patients to IO infiltrations of PRP, knowing the biological mechanisms behind this improvement is essential in advancing the development of more effective therapies. Aberrant behaviour of subchondral bone MSCs with decreased proliferation and multipotency and increased osteoblastogenesis have been previously reported in OA animal models and in human studies [13,14,15,16]. Another parameter contributing towards the progression of OA is cellular senescence. The accumulation of senescent cells and the presence of senescence associated secretory phenotype (SASP) have also been described as features of OA [17]. The local clearance of senescent cells has been shown to create a pro-regenerative environment in the joint of post traumatic OA transgenic mice [18]. 

We therefore hypothesized that administering IO infiltrations of PRP combined with IA injections in hip OA patients enhanced their subchondral bone MSC proliferation capacity, reduced their senescence, and modified their gene expression in favour of MSC multipotency and supporting cartilage regeneration. The present work is therefore aimed to explore whether autologous PRP treatment had any stimulating effect on subchondral bone MSCs in hip OA patients.

## 2. Materials and Methods

### 2.1. Patients

The investigation was performed using subchondral bone marrow aspirate (BMA) samples from 12 patients with hip OA degrees 2 and 3 according to the Tönnis scale [19], and treated at the Arthroscopic Surgery Unit (UCA) with a combination of IO and IA PRP infiltrations. The autologous PRP infiltrations were performed as described in the below section and the time period between the two infiltrations was 14 days. Routine clinical follow-up at 6 months after the second infiltration was used to assess the patient’s response to treatment in this study. This follow-up included the clinical examination by the physician as well as the Hip dysfunction and Osteoarthritis Outcome Score (HOOS) completed by the patient at the different visits to the centre.

Positive response (referred to as ‘responders’ from now on) was defined according to the physician assessment and a reduction in the HOOS pain score of at least 10 points from the baseline (minimal clinically important improvement (MCII)) [20]. A HOOS pain score of less than 10 points was classified as a negative response (referred to as ‘non-responders’ from now on). The patients’ demographics, clinical details, their medication or their absence thereof along with their 6-month treatment response assessments are presented in the Table 1.

### 2.2. PRP Preparation and Administration Procedure

The PRP preparation process has been previously described [10]. Briefly, venous blood (72 mL) was withdrawn from patients in 9 mL sodium citrate 3.8% (*w/v*) tubes. This was centrifuged at 580 g for 8 min at room temperature (RT) to collect the 2 mL plasma fraction above the sedimented red blood cells (RBCs); care was taken to avoid the buffy coat layer. PRP was activated with calcium chloride 10% (*w/v*) just prior to infiltration.

The administration of PRP included three different injections in different anatomical locations performed in the operating room. First, one PRP IA injection was conducted, followed by two PRP IO injections, according to the technique described by Fiz et al. [11]. Briefly, sedation of the patient was induced with a single dose of normal saline solution as well as a single dose of midazolam (0.03–0.05 mg/kg) and fentanyl (3.2 mg/kg) in the peripheral vein; a single or repeated dose of propofol was also administered (1–2 mg/kg), depending on the duration of the infiltration. An IA injection guided by ultrasound was conducted using an 18-gauge needle oriented in the same direction as the anterolateral-distal arthroscopic portal. With a 30° of joint flexion to facilitate the infusion of the PRP infiltration, 8 mL of PRP was injected into the joint space.

Next, with the guidance of a fluoroscope, an anterior–posterior view of the hip joint was reached in order to perform the first IO infiltration into acetabulum. The trocar was placed in the cranial–caudal direction, parallel to the horizontal plane and at an inclination of 20°. Once the trocar was introduced into the lateral acetabular wall and situated 1 cm from the articular line, 5 mL of PRP was injected. Finally, the second IO injection was performed into the femoral head whose point of entry was situated 1 cm lateral to the sartorial muscle. The femoral head was approached at the union of the femoral neck and head, with the trocar orientated in the anterolateral–distal direction. The trocar was introduced 1 cm below from the articular surface, and 5 mL of PRP was injected. Prior to infiltration, 5 mL of BMA was collected from femoral head subchondral bone for this study and labelled as ‘Pre’ (before treatment). During the first few hours after treatment, assisted walking with crutches and a minimal initial load was recommended due to the intervention itself. The next day, the patient could bear weight and take analgesics (acetaminophen) as required for pain, with limited physical activity. The same procedure was performed 14 days after the first treatment, and another 5 mL of BMA was collected and labelled as ‘Post’ (after treatment). 

### 2.3. Bone Marrow Aspirate Sample Processing and the Establishment of MSC Cultures

All BMA samples (5 mL) were processed as previously described [21] Following the lysis of RBCs using ammonium chloride, and after washing the cell pellet twice with 10 mL of PBS, the nucleated cells were re-suspended in complete MSC media (StemMACS™ MSC Expansion media, Miltenyi Biotec, Bisley, UK) in a volume equivalent to the original BMA, then seeded in a 6-well plate (Corning, New York, NY, USA ) at 750 µL/well containing 1 mL of StemMACS™ media to enable MSC attachment. After 48 h, the media were changed to low glucose DMEM supplemented with antibiotics and 10% human serum (Sigma-Aldrich, Dramstadt, Germany) for MSC expansion to passage 1 (p1). We have previously shown that the expansion of MSCs, particularly in FCS-containing media, affects their colony characteristics and gene expression profiles, while the use of human serum leads to a better preservation of their native characteristics [22,23]. Half media changes were performed twice weekly, and the cells were trypsinised and expanded when approximately 60–70% confluent. P1 MSCs were then frozen at −80 °C in aliquots and used for further analysis, as described below.

### 2.4. Colony-Forming Unit-Fibroblast (CFU-F) Assay and Colony Analysis

The colony-forming unit-fibroblast (CFU-F) assay was used to quantify the highly-proliferative colony-forming cells in p1 cultures [24] and to compare their proportions in Pre- and Post-p1 MSC cultures. For the assay, the frozen vials were first defrosted and revived in StemMACS™ media with no further culture expansion. Cell seeding densities for the CFU-F assay were optimised using 1000, 2000, and 5000 cells/100 mm per Petri dish (Corning) from three randomly selected p1 cultures to generate a minimum of 30 colonies/dish for the quantification of colonies. Based on these optimisation results, all of the remaining p1 cultures were seeded at 5000 cells/dish and the data were presented relative to 5000 seeded cells. In brief, 5000 cells were seeded in duplicate in the 100 mm Petri dishes in 10 mL of StemMACS™ media for 48 h in an incubator at 37 °C and 5% CO_2_. After 48 h, StemMACS™ media were aspirated and the dish was washed with 10 mL of PBS. After the wash, 10 mL of DMEM (ThermoFisher, Waltham, MA, USA) containing 10% human serum (DMEM + HS) was added to each of the Petri dishes and half of the media were changed once a week. On day 14, the media were aspirated, the colonies were fixed using 3.7% formaldehyde (Fisher Scientific, NewHampshire, USA), and stained with 1% (*w/v*) methylene blue (Sigma-Aldrich). A colony was defined as consisting of at least 50 cells. After staining, the dishes were scanned, and the number of colonies were counted and averaged followed by investigating the colony areas [14].

Colony areas are considered as indicative of the proliferative capacities of colony-forming MSCs [25,26]. In brief, scanned images were analysed using ImageJ software (version 1.53q, NIH). The images were converted to ‘grey scale’ and all of the colonies were given an outline manually; colony areas were calculated by the software based on a 100 mm (dish diameter) scale. The analysis was performed on the Pre and Post cultures and subsequently correlated with donor age, treatment response, and other laboratory measurements.

### 2.5. Senescence Assay

The accumulation of senescent cells including MSCs is considered as one of the cellular mechanisms behind OA pathology [17,18]. To measure the proportions of senescent cells in p1 Pre and Post MSC cultures, the gold-standard histochemical senescence-associated beta galactosidase (SA-β-gal) assay was performed according to the manufacturer’s protocol (Sigma-Aldrich), as previously described [15,27]. P1 MSCs were defrosted and 4 × 10^4^ cells from each sample were plated in duplicate wells of a 6-well plate overnight for adhesion in 1.5 mL of StemMACS™ media. The next day, the media were aspirated and cells were washed in PBS, then 1.5 mL of fixation buffer was added to the plates and incubated for 6–7 min at RT. The cells were then rinsed three times with PBS and stained using 1 mL of the staining mixture prepared as per the manufacturer’s protocol. The dishes were then sealed in parafilm and incubated at 37 °C without CO_2_ overnight. The following day, the cells were observed for the presence of blue (or SA-β-gal positive cells) under light microscopy. A minimum of 100 total cells/well were counted by three independent observers (PG, EJ, and HO); the data were averaged, and the percentage of blue stained cells was presented as the percentage of total counted cells.

### 2.6. Gene Expression

The study of gene expression (GE) in p1 MSCs was performed in order to evaluate any transcriptional changes in the cells related to proliferation/senescence, multipotentiality, osteo- and chondrogenesis as well as cartilage remodelling and support for cartilage anabolism [26]. For GE, revived p1 cells were lysed using lysis buffer (Norgen Biotek, Ontario, Canada) and the cell lysate was frozen at −80 °C. RNA extractions for all samples were performed as per the manufacturer’s protocol using columns provided by the manufacturer. RNA quantity and quality was assessed using a Nanodrop spectrophotometer. The RNA was then frozen at −80 °C until cDNA preparation. cDNA was prepared by adding the reverse transcriptase master mix (Fluidigm) to the RNA samples in the thermocycler (5 min at 25 °C, 30 min at 42 °C, 5 min at 85 °C, and then held at 4 °C). On completion, the cDNA was pre-amplified [26,28] with pooled Taqman assays and the pre-amplification (PA) reaction mix using a 14-cycle protocol (2 min at 95 °C, 15 s at 95 °C, 4 min at 60 °C, and then held at 4 °C). Finally, the PA samples were added to the 48.48 GE Fluidigm Integrated Fluid Circuit (IFC) along with 48 Taqman probes (listed in Supplementary Table S1) and loaded onto the Biomark platform as per the manufacturer’s instructions. *HPRT1* was used as a reference gene.

### 2.7. Statistical Analysis

All data were analysed using Graph Pad Prism software (version 9.0). For any given set of data, Gaussian distribution was first tested using the Shapiro–Wilk and the Kolmogorov–Smirnov tests for normality. Paired tests (Pre versus Post) were used to analyse the data where possible, otherwise non-parametric tests were used. The Spearman correlation test was used to study the correlations with patient ages.

## 3. Results

### 3.1. Patients Characteristics and Response to Treatment

Twelve patients diagnosed with hip OA participated in this study. Of these, four were men and eight were women and their median age was 59.5 years old (range 40–86) (Table 1). Seven out of 12 patients responded well to treatment and five patients were considered non-responders (Table 1). Among the males, the response rate was 50% (2/4). In females, the response rate was 62.5% (5/8). The Tönnis score of all patients ranged between 2 and 3, irrespective of the treatment response. When the cohort was split based on age (using 60 years old as a cut-off point), the response rate in the older group was 66.7% (4/6) compared to 50% (3/6) in the younger patients. There were very few patients with concomitant medication, as seen in Table 1. COX-2 inhibitors were not taken as it is recommended not to take them during this PRP treatment.

### 3.2. Platelet-Rich Plasma Characterization

The median PRP platelet concentration was 355.5 × 10^3^ platelets/µL (CI: 276 to 398), with a concentration factor of 1.96 (CI: 1.83 to 2.14) above the baseline. Negligible amounts of RBCs or leukocytes were present (Table 2) and the PRP was classified as 13-00-11 according to the latest coding system for PRP studies [4].

### 3.3. Colony Numbers and Characteristics

**Figure 1:**
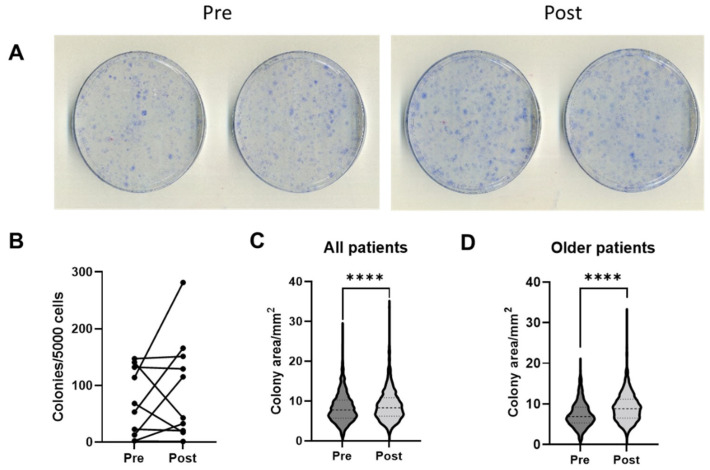
**Colony-forming capacity in p1 MSCs before (Pre) and 2 weeks after (Post) PRP infiltrations** (n = 10 patients, symbols represent averages of the duplicate dishes). (**A**) Images of duplicate dishes from a representative patient. (**B**) CFU-F frequencies in the Pre and Post samples. (**C**) Violin plots showing the colony area distributions in all patients. (**D**) Violin plots showing the colony area distributions in older (>60 years old) patients. **** *p* < 0.0001, Mann–Whitney U test.

Paired colony data were available for 10 patients. On completion of the 14-day culture period, the colonies were fixed, stained, counted, and averaged for duplicate dishes (Figure 1A). There were no significant differences in the number of colonies per 5000 seeded cells between the Pre and Post MSCs (Figure 1B) and no correlations were found between the proportions of colonies and donor age for both the Pre and Post samples (Supplementary Figure S1).

After the analysis of colony numbers, colony areas that are indicative of the proliferative capacity of individual MSCs [23,25] were analysed using ImageJ software, version 1.53q, NIH, Bethesda, MD, USA. Altogether, 1197 colonies from the Pre and 1370 colonies from the Post samples were analysed, and the average colony area was found to be significantly 14.5% higher (*p* < 0.0001) in the Post samples (median = 8.30 mm^2^) compared to the Pre samples (median = 7.74 mm^2^) (Figure 1C). Interestingly, an even larger (28.2%) increase in colony areas was observed in older patients (>60 years old), from a median of 6.87 mm^2^ in the Pre samples to a median of 8.81 mm^2^ in the Post samples, *p* < 0.0001, Figure 1D). In agreement, in the Pre samples, colony areas displayed a trend for a decrease in older patients, whereas this trend was no longer evident in the Post samples (Supplementary Figure S1). Altogether, these data indicated an increase in the MSC proliferative capacity after PRP infiltrations, particularly in older patients.

### 3.4. MSC Multipotentiality Marker Expression and Proportion of Senescent Cells

**Figure 2:**
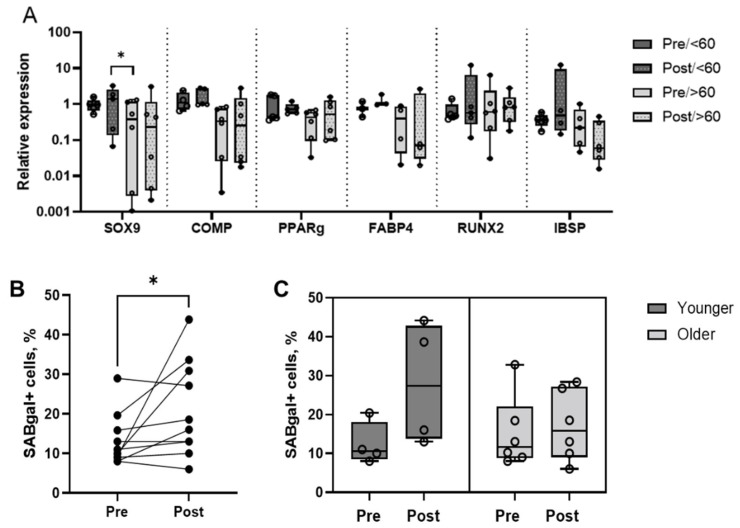
The MSC multipotentiality marker expression and the proportions of senescent cells in p1 MSCs before (Pre) and 2 weeks after (Post) PRP infiltrations. (**A**) Relative expression of *SOX9*, *COMP, PPARγ, FABP4, RUNX2*, and *IBSP* in the Pre and Post samples segregated by age. (**B**) Comparison of senescent cell percentages in the Pre and Post samples (all patients). (**C**) Comparison of the senescent cell percentages in the Pre and Post samples (patients segregated by age). Circular symbols in all panels represent individual patients, horizontal bars on C represent medians, * *p* < 0.05, Wilcoxon rank sum test.

Next, the MSC multipotentiality marker expression and the proportions of senescent cells in p1 cultures were explored (Figure 2). MSC multipotentiality gene expression was measured by qPCR for *SOX9* (*Sex determining region Y box 9*) and *COMP* (*Cartilage Oligomeric Matrix Protein*) (chondrogenesis markers), *PPARγ* (*Peroxisome Proliferator Activated Receptor Gamma*) and *FABP4* (*Fatty Acid Binding Protein 4*) (adipogenesis markers), and *RUNX2* (*Runt related transcription factor 2*) and *IBSP* (*Integrin binding sialoprotein*) (osteogenesis markers) [29] (Figure 2A). As expected, trends for the lower expression of all multipotentiality genes were seen in older patients (>60 years old). There was a significant increase in the level of *SOX9* expression in the Post samples in younger patients compared to the Pre samples in the older patients (Figure 2A). Overall, these data indicated that p1 cultures from hip OA patients expressed MSC multipotentiality genes, but PRP infiltrations only induced small changes in the levels of their expression.

Senescence is the termination of the proliferative capacity of cells and the accumulation of senescent cells has closely been associated with OA, so targeting cellular senescence represents an attractive therapeutic option [17,18]. Therefore, the level of senescence in p1 MSCs was next investigated to explore any effect of the PRP infiltrations on the senescence process (Figure 2B,C).

Unexpectedly, the proportions of senescent cells were found to be significantly increased in the Post samples in comparison to the Pre samples (*p* = 0.043) (Figure 2B), but not in all patients. Rather than a direct effect of PRP infiltrations, senescence increases in some patients could have been a result of a rapidly progressing OA process in the affected joint. As senescence is closely associated with advancing age [30,31], the data were next segregated on the basis of the patients’ age, and while no statistically significant differences were found, the increase in senescent cells in the Post samples was less prominent in the older patients compared to the younger patients (Figure 2C). On this basis, it could be hypothesised that PRP treatment might have provided some degree of the ‘resistance’ of MSCs to the OA-driven senescence process, and that it might be more effective in older patients. 

### 3.5. MSC Changes in Relation to Treatment Response

As seen above, MSCs from older patients (>60 years old) appeared to respond better to PRP, at least in the CFU-F and senescence assays, compared to younger patients. Next, any differences in MSC behaviour were analysed in relation to the patients’ responses to therapy. The response was evaluated at 6 months after the second injection and was based on HOOS scoring and clinical examination conducted by a physician at the 6-month follow up time point (Table 1). Seven out of 12 patients (four older, three younger) were considered good responders, the other five (two older, three younger) were defined as non-responders.

**Figure 3:**
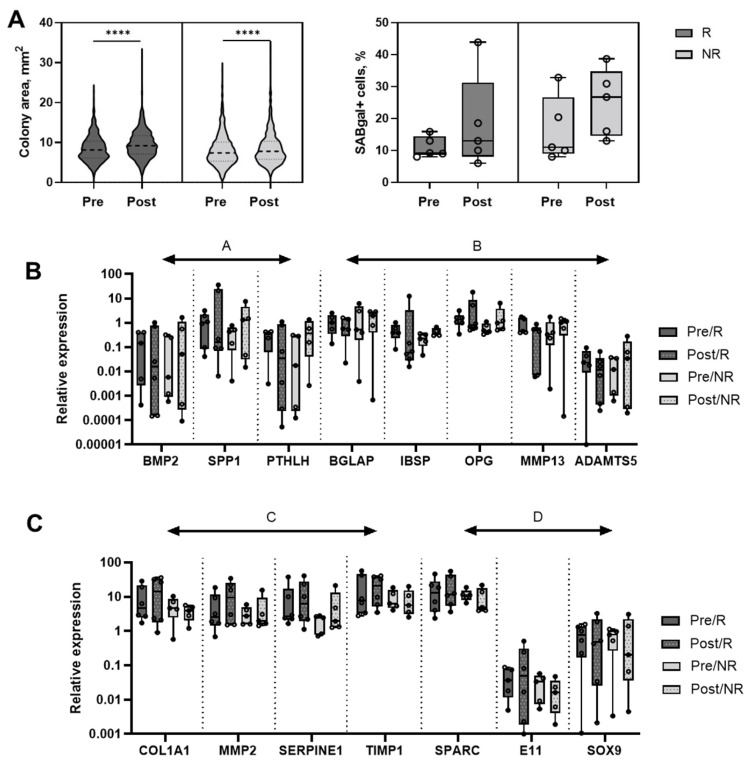
**The MSC changes in relation to treatment response.** (**A**) Colony area comparison as observed in the Pre and Post p1 MSCs (left panel) and percentages of senescent cells in the Pre and Post p1 MSCs (right panel) segregated based on response to treatment. (**B**,**C**) Relative expression of *BMP2, SPP1, PTHLH, BGLAP, IBSP, OPG, MMP13, ADAMTS5* and *COL1A1, MMP2, SERPINE1, TIMP1, SPARC, E11* and *SOX9* in p1 MSCs segregated based on response to treatment. Circular symbols in each panel represent individual patients, horizontal bars represent medians, **** *p* < 0.0001, Mann–Whitney U test.

Comparing the CFU-F assay results between the responders and non-responders, the colony area increases were more prominent in the responders (13.2% increase, from a median of 8.12 mm^2^ in Pre samples to a median of 9.19 mm^2^ in Post samples) compared to the non-responders (5.1% increase, from a median of 7.39 mm^2^ in Pre samples to a median of 7.77 mm^2^ in the Post samples, *p* < 0.0001) (Figure 3A). For the senescence assay, the segregation of data between the responders and non-responders indicated a lesser increase in the senescent cells in the Post samples in responders compared to the non-responders, but the differences failed to reach statistical significance (Figure 3B). 

Gene expression changes were next evaluated separately for the responders and non-responders (Table 3) and any genes with more than 2-fold differences between the Pre and Post in either group were analysed in more detail (Figure 3C). Any genes for which the detection was found to be highly donor variable and very low (i.e., expression detected in less than 50% of samples) [26,32] were eliminated from the analysis. Four groups of molecules could be identified based on different patterns of expression (Figure 3C). In group A (*BMP2, SPP1, PTHLH*), the gene expression changes in the non-responders were in the opposite direction to the responders, a >2-fold increase in the non-responders, and a >2-fold decrease in responders. In the group B genes (*BGLAP, IBSP, OPG, MMP13, and ADAMTS5*), a similar ‘opposite direction’ pattern was observed, however, the >2-fold changes were seen only in one group of patients (mostly increased non-responders). The behaviour of the group C genes (*COL1A1, MMP2, SERPINE1, TIMP1*) was similar to the group B genes but in contrast to the group B genes, they was a >2-fold increase in the responders (Figure 3C). Finally, the group D genes (*SPARC, E11, SOX9*) were >2-fold reduced in the non-responders but not in the responders (Figure 3C). Although no statistically significant differences could be found due to the small group sizes, differentially-expressed genes could be identified and interestingly, most of these genes belonged to the osteogenesis, chondrogenesis, and cartilage homeostasis categories. Altogether, these data suggest that MSC gene expression changes following PRP infiltrations could be partly contributing to inducing joint tissue responses, in addition to a significant enhancement in the MSC proliferation seen in the CFU-F assay.

## 4. Discussion

Joint-sparing interventions such PRP IA injections are becoming popular in OA management [33]. The biological mechanisms behind PRP action and their links to improvements in clinical outcomes remain poorly understood but are believed to relate to both the PRP cellular composition and host (patient-related) factors [34,35,36]. In comparison to PRP joint injections, in which PRP primarily acts on the cartilage and synovium, IO PRP infiltrations provide an advantage of better access to the subchondral bone and bone–cartilage interface, potentially targeting the whole spectrum of OA-affected tissues. Furthermore, there is a possibility of a longer-term retention of PRP or its constituents in the target tissues, in contrast to the joint fluid that is likely to exchange its constituents with plasma more rapidly during locomotion.

In this study, we investigated the effect of autologous PRP on MSCs resident at the site of an IO PRP infiltration in patients with hip OA. BMAs were obtained before and 2 weeks after the first infiltration and MSCs were minimally-cultured in media containing human serum, which is known to better preserve MSC native characteristics [22,37]. This, and the fact that the study also investigated the *in vivo* effect of PRP in relation to clinical outcomes, makes our study very novel. To study the effects of PRP on MSC proliferation, we compared the number of colonies and colony areas in early-passage MSC cultures. We also investigated 47 transcripts associated with various MSC functions including osteogenesis and bone remodelling, chondrogenesis and cartilage homeostasis, adipogenesis, MSC support for angiogenesis as well as cell cycle and senescence-related molecules. As OA is an age-related disease and ageing is closely associated with cellular senescence [17,30,31], we evaluated the percentages of the senescent cells in MSCs following infiltrations. Finally, keeping the former in mind, we separated the data based on patient age and reported the clinical responses for each of these evaluations to further dissect the findings.

We first investigated any changes in MSCs at the single-cell level using a well-established CFU-F assay [25]. While we found no significant differences with respect to the number of colonies formed in the Post samples, the colony areas in the Post samples were found to be significantly higher compared to the Pre samples. This is indicative of the higher rates of proliferation of single MSCs post the PRP infiltrations. These results are also in line with one of our previous findings wherein we also found a significant increase in the colony area of MSCs from the synovial fluid (SF) following knee joint distraction (KJD) surgery, another joint-sparing intervention for OA [26]. In agreement, another clinical study described an enhancement of the *in vivo* MSC proliferation following autologous PRP injections [38] into the iliac crest, which coincided with an increase in the systemic and local concentrations of the PDGF-BB and FGF-2 growth factors known to be mitogenic for MSCs. Watt and co-workers also demonstrated an increase in the local FGF-2 in the synovial fluid 6 weeks post joint distraction [39]. While this is suggestive of the potential role of FGF-2 in mediating this effect, it remains to be further explored.

It is noteworthy that we found higher rates of response and better increases in the colony areas in older patients compared to all patients, suggesting that clinically, the older patients’ MSCs might respond better to PRP infiltrations than the MSCs from the younger patients. This is in line with our previous study when we investigated the responses of young- and old-donor MSCs to ‘young’ human serum and found that colony area increases were more pronounced in older donors [23]. These findings suggest that older individuals might gain more benefit from PRP treatments, and that the administration of allogeneic ‘young’ donor PRP may be considered as a future step. Indeed, IO injections of PRP from young rats prevented age-related bone degeneration in old rats in a pre-clinical study [40]. Interestingly, older donor MSCs also appeared to resist the accumulation of senescent cells better than the younger donor MSCs. Even though the data did not reach statistical significance, this presents a further insight into the potentially better effectiveness of PRP treatments in older patients. The MSC response after intraosseous PRP infiltration may be similar to the effect of cell therapies, in which MSCs from different niches are implanted with the intention of harnessing the therapeutic properties of these cells [41]. Thus, the in situ stimulation of MSCs by PRP may favour their “therapeutic effect”, considering they offer a cost effective and less invasive process than cell therapies with fewer regulatory limitations [42,43].

Finally, when we compared the data in terms of the response to treatments, we found that the MSC colony area increases after PRP infiltrations were stronger in the responders compared to the non-responders and that the responders’ MSCs also appeared to better resist the accumulation of senescent cells compared to the non-responders, suggesting that the treatment potentially activated mechanisms that helped these cells abstain from becoming senescent [44,45]. In terms of gene expression, the responder and non-responder MSCs tended to differ in the expression of genes associated with bone formation and cartilage turnover including osteoblast markers, matrix metalloproteinases, and their inhibitors. However, the differences failed to reach statistical significance due to the small group sizes. While these molecules represent good candidates to take onto a larger study, other relevant pathways should also be considered. For example, a recent study investigating the *in vitro* responses of macrophages and synovial fibroblasts to PRP in patients with knee OA demonstrated that the pathways most significantly affected by PRP in fibroblasts, the cells the closest in nature to MSCs, were related to the cell cycle, DNA synthesis, and cell survival [34]. Future investigations should therefore investigate larger panels of genes, encompassing many more markers of cell proliferation, survival, and apoptosis as well as assess many inflammation-driven signalling pathways that might be activated in MSCs from OA joints.

## 5. Conclusions, Limitations and Future Directions

Overall, we present the first-in-human evidence of subchondral bone MSC responses to IO PRP infiltrations. We also combined this evidence with the clinical responses of the patients to correlate the impact of PRP infiltrations on MSCs from OA patients, making this a novel study. Our data point towards better MSC responses to this treatment in older patients compared to the younger patients. Notably, combined IO and IA PRP infiltrations boosted the subchondral MSC proliferative and stress-resistance capacities, especially in patients over 60 years old. In terms of gene expression, there were no differences that were statistically significant. However, trends of gene expression within the defined groups between the responders and non-responders were intriguing and merit further research in the field.

We acknowledge that our sample size was limited, particularly following segregation into the different groups, but we used human patient samples from the site of infiltration reflecting as closely as possible the conditions of MSCs in their native target tissue. It should also be noted that, due to methodological limitations, the impact measured is for half of the treatment (one infiltration) and not the full treatment (two infiltrations). Thus, it is possible that the final effect will be more pronounced due to repeated injections [46], although further research is needed to validate this. 

Future investigation involving the use of uncultured MSCs in a larger patient group should give a better indication of the magnitude and clinical relevance of differences identified in the present work. In vivo, MSCs do not exist in isolation and co-cultures with other joint-resident cells may aid in uncovering other cellular and molecular mechanisms of PRP action *in vivo*, leading to potential ‘treatment response’ biomarkers to PRP administrations. Moreover, based on our findings, the future study of an ‘anti-ageing’ function of PRP, performed using direct comparisons between autologous and allogenic PRP treatments, deserves special attention. Furthermore, adapting techniques such as next-generation sequencing (NGS) present tremendous potential and novelty in terms of uncovering these mechanisms, especially considering the anti-ageing function of PRP on MSCs [47]. Altogether, our study has presented evidence that is the first of its kind to assess the impact of IO PRP infiltrations on MSC function in OA patients. At the same time, this work has paved the way for future research to further dissect the mechanisms involved in the same with the aim to enhance treatment for OA patients.

## Figures and Tables

**Table 1:** The patient demographics and clinical assessment before and after treatment.

Gender	Age, Years	Response	Tönnis	Medications
M	58	Positive	3	Unspecified treatment for arterial hypertension
F	61	Positive	2	None
F	56	Negative	3	Unspecified treatment for arterial hypertension
F	54	Negative	3	None
F	61	Positive	2	None
F	70	Positive	2	Thyroxine
M	61	Negative	2	Antihypertensives; Statins
M	40	Negative	2	None
F	55	Positive	3	None
F	64	Positive	3	None
M	56	Positive	3	Leflunomide
F	86	Negative	3	Escitalopram; Paracetamol

M: Male; F: Female.

**Table 2:** The characteristics of patient derived PRP application.

**PRP Preparation**	
Blood volume taken	72 mL
Anticoagulant	Sodium citrate 3.8% (*w/v*)
System	Close
Centrifugation	Once, 580 g/8 min
Final PRP volume	10 mL (IO) plus 8 mL (IA)
**PRP Characteristics**	
PRP Type	13-00-11
MPV	10.1 fL (CI: 9.30–10.50)
Red Blood Cells	<0.01 × 10^6^/μL
White Blood Cells	<0.05 × 10^6^/μL
Activation	CaCl_2_ 10% (*w/v*)
**Application Characteristics**	
Formulation type	Liquid
Administration route	IO plus IA
Dosage	2 infiltrations at two weeks interval
Volume	IO: 10 mL (5 mL acetabulum + 5 mL femoral head) IA injection: 8 mL
Dose (range of platelets)	IO injection: 2.76 × 10^9^–3.98 × 10^9^ IA injection: 2.21 × 10^9^–3.18 × 10^9^
Tissue	Cartilage, synovium, subchondral bone
Pathology	Hip osteoarthritis

PRP: platelet-rich plasma; IA: intraarticular; IO: intraosseous; MPV: mean platelet volume.

**Table 3:** A list of the genes with median values and fold differences between the Pre and Post samples in the responders and non-responders.

Genes	Median Relative Expression Values				Fold Differences, Post/Pre	
	Pre/R	Post/R	Pre/NR	Post/NR	R	NR
*RUNX2*	0.5016	0.6905	0.5779	0.8294	1.376595	1.435196
*ALP*	0.5477	0.9033	0.3409	0.5918	1.649261	1.735993
*IBSP*	0.3944	0.05968	0.22	0.3793	0.151318	1.724091
*COL1A1*	4.653	14.38	4.677	3.967	3.090479	0.848193
*BGLAP*	1.02	0.57	0.5216	1.952	0.558824	3.742331
*SPP1*	1.001	0.1541	0.4335	1.323	0.153946	3.051903
*SPARC*	12.91	11.64	11.16	4.895	0.901627	0.43862
*OPG*	1.34	0.8102	0.4803	0.9831	0.604627	2.046846
*ANKH*	0.4462	0.5939	0.5231	0.5771	1.331017	1.103231
*GREM1*	0.555	0.9183	0.3949	1.152	1.654595	2.917194
*E11*	0.03657	0.04949	0.03373	0.01627	1.353295	0.48236
*BMP2*	0.1449	0.01558	0.005887	0.05139	0.107522	8.729404
*PTHLH*	0.3283	0.03508	0.01772	0.3664	0.106853	20.6772
*SOX9*	0.7716	0.4732	0.7836	0.2052	0.613271	0.261868
*COMP*	0.6122	0.7304	0.6867	1.027	1.193074	1.495558
*ACAN*	0.9876	1.029	0.6267	1.112	1.04192	1.774374
*MMP1*	0.1341	0.4476	0.06261	0.1486	3.337808	2.373423
*MMP2*	2.528	9.414	2.76	1.982	3.723892	0.718116
*MMP13*	0.4501	0.4859	0.3275	1.014	1.079538	3.096183
*ADAMTS4*	0.02331	0.01155	0.01217	0.03424	0.495495	2.813476
*SERPINE1*	2.658	6.356	2.449	1.942	2.391272	0.792977
*TIMP1*	7.729	20.88	6.346	5.652	2.701514	0.89064
*TIMP2*	2.32	3.828	2.023	2.37	1.65	1.171527
*TIMP3*	4.378	7.125	2.982	1.84	1.627455	0.617036
*PTGS2*	1.096	0.8219	1.066	1.071	0.749909	1.00469
*PPAR-ɣ*	0.431	0.6278	0.5237	0.7886	1.456613	1.505824
*CXCL12*	1.246	1.911	1.721	2.398	1.533708	1.393376
*VEGFA*	1.099	2.048	1.109	1.403	1.863512	1.265104
*VEGFC*	6.912	8.79	7.783	8.161	1.271701	1.048567
*PDGFRB*	1.009	1.253	0.6647	0.9144	1.241824	1.375658
*EGFR*	0.06623	0.08422	0.03631	0.1435	1.271629	3.952079
*FGFR1*	0.387	0.571	0.2379	0.4063	1.475452	1.70786
*FGFR2*	0.2632	0.1553	0.3359	0.3029	0.590046	0.901756
*TGFBR2*	0.4042	0.8372	0.3433	0.4072	2.071252	1.186135
*PTPRC*	0.6435	1.469	0.5147	0.6744	2.282828	1.310278
*NT5E*	1.536	1.891	1.718	2.714	1.23112	1.579744
*Thy1*	1.97	1.764	1.33	1.625	0.895431	1.221805
*P21*	1.215	1.097	1.091	0.9113	0.902881	0.835289
*P53*	0.646	0.6835	0.5047	0.5311	1.05805	1.052308
*P16*	1.705	1.359	1.483	2.114	0.797067	1.425489
*HPRT1*	1	1	1	1	1	1

R = responders and NR = non-responders.

## Data Availability

The data used to support the findings of this study are available from the corresponding author upon reasonable request.

## Supplementary Materials

The following supporting information can be downloaded at: https://www.mdpi.com/article/10.3390/jcm11133891/s1, Figure S1: Correlation of CFU-F frequencies. Table S1: List for genes and probes for qPCR.
