# Supplementary material for: Effect of Combined Intraosseous and Intraarticular Infiltrations of Autologous Platelet-Rich Plasma on Subchondral Bone Marrow Mesenchymal Stromal Cells from Patients with Hip Osteoarthritis
Source: J Clin Med. 2022 Jul 4;11(13):3891. doi: 10.3390/jcm11133891 (PMC9267269; doi:10.3390/jcm11133891)
Supplement: Supplementary file 1 [file jcm-11-03891-s001.zip › jcm-1763325-supplementary.pdf]

Supplementary Figure S1

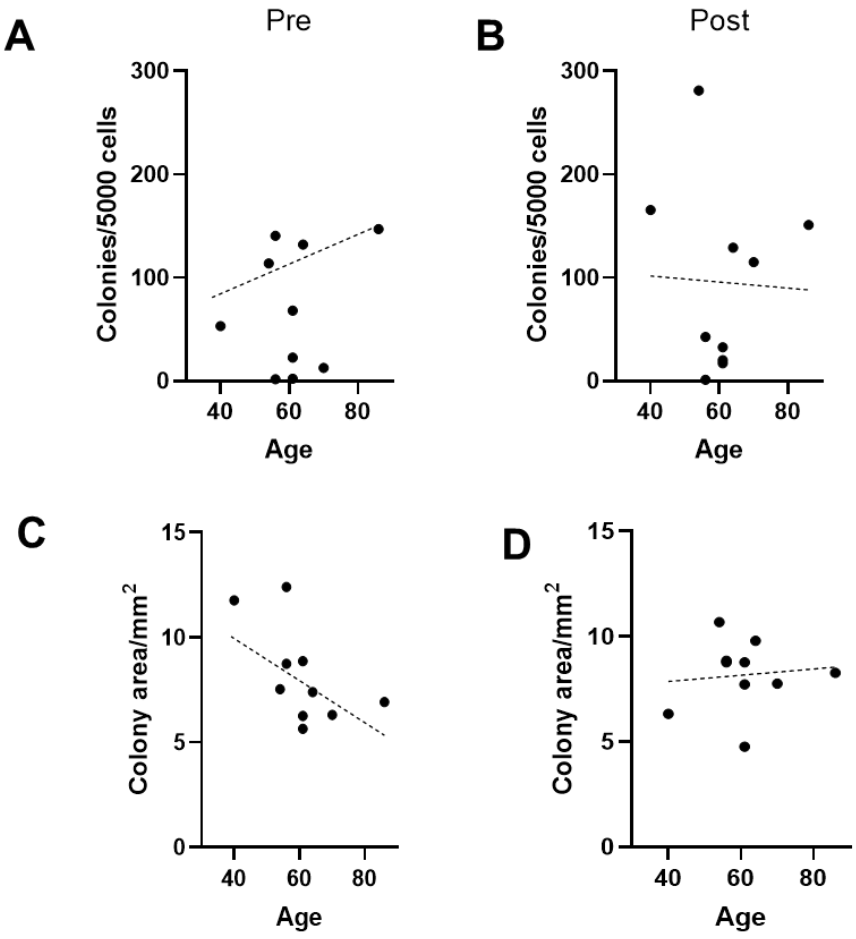

***Correlation of CFU-F frequencies**: **(A)** Correlation of CFU-F frequencies per 5000 MSCs with advancing age in the pre samples (R=0.258) **(B)** Correlation of CFU-F frequencies per 5000 MSCs with advancing age in the post samples (R=0.039) **(C)** Correlation of CFU-F colony areas with advancing age in the pre samples (R=0.596) **(D)** Correlation of CFU-F colony areas with advancing age in the post samples (R=.104)*

Supplementary table S1: List for genes and probes for qPCR

No	Gene Name	Gene ID	Assay	Category
1	Runt related transcription factor 2	RUNX2	Hs00231692_m1	Osteogenesis and bone remodelling
2	Alkaline phosphatase	ALP	Hs00758162_m1	
3	Integrin binding sialoprotein	IBSP	Hs00173720_m1	
4	Collagen Type I Alpha 1 Chain	COL1A1	Hs01076777_m1	
5	Bone Gamma-Carboxyglutamate Protein	BGLAP	Hs01587814_g1	
6	Secreted Phosphoprotein 1	SPP1	Hs00959010_m1	
7	Secreted Protein Acidic And Cysteine Rich	SPARC	Hs00277762_m1	
8	Receptor activator of nuclear factor kappa-B Ligand	RANKL	Hs01092186_m1	
9	Osteoprotegerin	OPG	Hs00900360_m1	
10	Human homolog of the murine progressive ankylosis gene	ANKH	Hs01064613_m1	
11	Gremlin 1, DAN Family BMP Antagonist	GREM1	Hs00171951_m1	
12	Podoplanin	PDPN/E11	Hs00366766_m1	
13	Bone Morphogenetic Protein 2	BMP2	Hs00154192_m1	
14	Osteomodulin	OMD	Hs00192325_m1	
15	Parathyroid Hormone Like Hormone	PTHLH	Hs00174969_m1	Chondrogenesis and cartilage homeostasis
16	Sex determining region Y box 9	SOX9	Hs00165814_m1	
17	Cartilage Oligomeric Matrix Protein	COMP	Hs00164359_m1	
18	Aggrecan	ACAN	Hs00153936_m1	
19	Matrix Metalloproteinase 1	MMP1	Hs00899658_m1	
20	Matrix Metalloproteinase 3	MMP3	Hs00968308_m1	
21	Matrix Metalloproteinase 13	MMP13	Hs00942589_m1	
22	Matrix Metalloproteinase 9	MMP9	Hs00957562_m1	
23	A disintegrin and metalloproteinase with thrombospondin motifs 4	ADAMTS4	Hs00192708_m1	
24	A disintegrin and metalloproteinase with thrombospondin motifs 4	ADAMTS5	Hs01095524_m1	
25	Collagen Type X Alpha 1 Chain	COL10A1	Hs00166657_m1	
26	Serpin Family E Member 1	SERPINE1	Hs00167155_m1	
27	Stathmin 2	STMN2	Hs00975800_m1	
28	Tissue inhibitor of metalloproteinase- 1	TIMP1	Hs00171558_m1	
29	Tissue inhibitor of metalloproteinase- 2	TIMP2	Hs01091319_m1	
30	Tissue inhibitor of metalloproteinase- 3	TIMP3	Hs00927214_m1	
31	Prostaglandin-Endoperoxide Synthase 2	PTGS2	Hs00153133_m1	
32	Peroxisome Proliferator Activated Receptor Gamma	PPAR-ɣ	Hs00602622_m1	Adipogenesis and stromal support for angiogenesis
33	Fatty Acid Binding Protein 4	FABP4	Hs00609791_m1	
34	C-X-C Motif Chemokine Ligand 12	CXCL12	Hs00171022_m1	
35	Vascular Endothelial Growth Factor A	VEGFA	Hs00900058_m1	
36	Vascular Endothelial Growth Factor C	VEGFC	Hs01099206_m1	

37	Platelet derived growth factor receptor beta	PDGFRB	Hs01019589_m1	MSC autocrine support
38	Epidermal growth factor receptor	EGFR	Hs01076078_m1	
39	Fibroblast growth factor receptor 1	FGFR1	Hs00241111_m1	
40	Fibroblast growth factor receptor 2	FGFR2	Hs01552926_m1	
41	Transforming growth factor beta receptor 2	TGFBR2	Hs00559661_m1	
42	Protein Tyrosine Phosphate Receptor type C	PTPRC	Hs00898487_s1	CD45
43	5'-Nucleotidase Ecto	NT5E	Hs00159686_m1	CD73
44	Thy-1 cell surface antigen	Thy1	Hs06633377_s1	CD90
45	Cyclin dependent kinase inhibitor 1A (AKA p21)	p21/CDKN1A	Hs00355782_m1	Senescence/cell cycle
46	Tumour protein 53	p53	Hs01034249_m1	
47	Cyclin dependent kinase inhibitor 2A (AKA p16)	p16/CDKN2A	Hs00923894_m1	
48	Hypoxanthine phosphoribosyl transferase 1	HPRT1	Hs99999909_m1	House-keeping gene
